# Predicting the serum digoxin concentrations of infants in the neonatal intensive care unit through an artificial neural network

**DOI:** 10.1186/s12887-019-1895-7

**Published:** 2019-12-27

**Authors:** Shu-Hui Yao, Hsiang-Te Tsai, Wen-Lin Lin, Yu-Chieh Chen, Chiahung Chou, Hsiang-Wen Lin

**Affiliations:** 1 College of Pharmacy, China Medical University, Taichung, Taiwan; 2 Department of Pharmacy, China Medical University Beigan Hospital, Yunlin, Taiwan; 3 Institute of Clinical Pharmacy and Pharmaceutical Science, National Cheng Kung University, Tainan, Taiwan; 4 Department of Pharmacy, National Cheng Kung University Hospital, Tainan, Taiwan; 5 Department of Pharmacy, China Medical University Hospital, Taichung, Taiwan; 6 Health Outcomes Research and Policy, Harrison School of Pharmacy, Auburn University, Auburn, AL USA; 7 Department of Medical Research, China Medical University Hospital, Taichung, Taiwan

**Keywords:** Artificial neural network, Infants, Patent ductus arteriosus, Digoxin concentration

## Abstract

**Background:**

Given its narrow therapeutic range, digoxin’s pharmacokinetic parameters in infants are difficult to predict due to variation in birth weight and gestational age, especially for critically ill newborns. There is limited evidence to support the safety and dosage requirements of digoxin, let alone to predict its concentrations in infants. This study aimed to compare the concentrations of digoxin predicted by traditional regression modeling and artificial neural network (ANN) modeling for newborn infants given digoxin for clinically significant patent ductus arteriosus (PDA).

**Methods:**

A retrospective chart review was conducted to obtain data on digoxin use for clinically significant PDA in a neonatal intensive care unit. Newborn infants who were given digoxin and had digoxin concentration(s) within the acceptable range were identified as subjects in the training model and validation datasets, accordingly. Their demographics, disease, and medication information, which were potentially associated with heart failure, were used for model training and analysis of digoxin concentration prediction. The models were generated using backward standard multivariable linear regressions (MLRs) and a standard backpropagation algorithm of ANN, respectively. The common goodness-of-fit estimates, receiver operating characteristic curves, and classification of sensitivity and specificity of the toxic concentrations in the validation dataset obtained from MLR or ANN models were compared to identify the final better predictive model.

**Results:**

Given the weakness of correlations between actual observed digoxin concentrations and pre-specified variables in newborn infants, the performance of all ANN models was better than that of MLR models for digoxin concentration prediction. In particular, the nine-parameter ANN model has better forecasting accuracy and differentiation ability for toxic concentrations.

**Conclusion:**

The nine-parameter ANN model is the best alternative than the other models to predict serum digoxin concentrations whenever therapeutic drug monitoring is not available. Further cross-validations using diverse samples from different hospitals for newborn infants are needed.

## Background

Digoxin is one of common medications used for pediatric heart failure [1], especially for premature infants or neonates with clinically significant patent ductus arteriosus (PDA), which is a condition where the ductus arteriosus fails to close after birth. While the potential biomarkers involved in PDA closure were suggested to be cyclooxygenase and peroxidase enzymes [2], indomethacin, or ibuprofen were the commonly used pharmacological interventions to stimulate PDA close in neonates [2]. In addition, digoxin is one of the agents to reduce PDA influence [2], and is particularly useful when natural closure of PDA, diuretics or nonpharmacological interventions fail, or when infants are unable to receive surgical treatment [2, 3]. With considerations of digoxin toxicity, and the impacts associated with concomitant metabolic abnormalities (i.e., hypokalemia) due to diseases or co-medications (i.e., indomethacin [4, 5]), lower value of trough serum digoxin concentration range (0.5–0.8 ng/ml) was preferred than the wider range (0.8 to 2.0 ng/ml) for those patients with chronic heart failure [6]. However, the preferred therapeutic range of digoxin in premature infants or neonates and its limited evidence regarding pharmacokinetic (PK) changes among these neonates, which were associated with various conditions (e.g., birth weight, gestational age, and organ maturity) [7, 8], remain the concerns in clinical practice.

The published guideline on pediatric dosing did recommend that drug dosing regimens should be modified on the basis of drug characteristics and normalized to body weight for small children, especially newborn infants [9]. Ideally, population PK studies can be a way of compensating for the small volumes of blood samples from pediatric patients. For instance, some studies in different countries did try to predict digoxin concentrations for pediatric patients by conducting population PK studies [10–14]. While checking digoxin concentrations and determining its PK parameters (i.e. digoxin clearance) after reaching the steady state is feasible for adult patients, it remains challenging to access blood samples for neonatal patients with developing total body weight and in critical ill. Limited evidence has addressed the correlations between PK parameters or digoxin dose and their therapeutic outcomes in neonates [7]. An alternative way to predict digoxin serum concentrations, other than conducting a PK study and drawing blood samples, for babies in neonatal critical care units will be of great value.

Suematsu et al. identified age and weight as two important factors for estimating digoxin clearance in pediatric patients [8]. Gender, digoxin dose, the presence of drug interactions, prematurity, and the presence of heart failure were not considered as impactful factors [8]. The authors applied traditional statistical approaches, assuming linear correlations existed between digoxin clearance and age, weight, dose, gender, and other variables, and a normally distributed digoxin concentration in this study. However, the assumptions of linear correlations, a normal distribution, and multicollinearity are usually violated in a complex biological system. Instead, the other recommended approaches to better capture non-linear relationships and the existence of multi-collinearity between drugs and patient characteristics in the complex human body, especially for infants, is artificial neural network (ANN) modeling [15].

ANN modeling have been increasingly applied in a variety of pharmaceutical science research predicting drug discovery, medical diagnoses, or clinical outcomes (e.g., mortality) [16–19]. These studies focused on adult patients, and provided limited evidence on the efficacy and safety of the treatments for pediatric patients, in particular neonates. For instance, Hu et al. applied machine learning techniques to predict the initial digoxin dosage in adult patients using the independent variables gender, age, weight, serum digoxin concentration, liver function, serum creatinine, blood *urea* nitrogen, albumin, potassium, and congestive heart failure diagnosis for adult patients with serum digoxin concentrations within the normal range (i.e., 0.5 to 0.9 ng/ml) [19]. Although this study found that the initial digoxin dose could be predicted accurately with ANN techniques [19], their findings cannot be applied to neonates directly, especially to those who are treated in critical care units. To date, only few ANN models have been applied with biological relevance to newborn fetal growth [20] and for survival prediction in pediatric trauma patients [21] and preterm birth [22]. Thus, the objective of this study was to compare and contrast the predicted concentrations of digoxin estimated from traditional regression modeling and from ANN modeling for the critically ill newborn infants prescribed with digoxin for clinically significant PDA in order to facilitate further medical decisions about the effectiveness, side effects, and concentrations of digoxin for such tiny critically ill patients in the future.

## Methods

### Subjects and data

A retrospective medical chart review using data from routine clinical practice was conducted to prepare the datasets for modeling and validation, respectively (Approval by Institutional Review Board [CMUH107-REC3–083]). Newborn infants who were taken care for in the Neonatal Intensive Care Unit (NICU) of China Medical University (CMU) Children’s Hospital and given digoxin (i.e., expected to reach acceptable therapeutic range) due to PDA between April 1, 2013 to April 30, 2017 were included in the modeling dataset. Their corresponding data were retrieved and managed for the model training. Because indomethacin was no longer available in Taiwan since year of 2010 and some evidences showed that indomethacin could increase digoxin’s serum concentrations [4, 5], ibuprofen was chosen as the first line to manage PDA closure for the infants in NICU of CMU Children’s Hospital. However, some patients who required the fluid restriction and/or had contraindications to use ibuprofen (e.g., gastrointestinal hemorrhage) were not appropriate to be prescribed with ibuprofen following the practice protocol in this unit. Further, those who were admitted to NICU between May 1, 2017 and December 31, 2017, and were prescribed with digoxin to deal with the PDA effect on heart and having observed serum digoxin concentrations within the acceptable range were identified as the samples for the model validation, whereas their data were managed in the validation dataset.

Ideally, the serum digoxin concentrations should be taken before the next dose or 8–24 h after the prior dose, and were evaluated for free form concentrations using homogeneous particle enhanced turbidimetric inhibition immunoassay (PETINIA). The acceptable digoxin therapeutic range was set up as 0.8 to 2.0 ng/ml for the management of heart failure or atrial fibrillation, where the concentrations equal or above 2.5 ng/ml for adults and 1.5 ng/ml for pediatric patients were considered as risk critical values, respectively. Such concentration values warrant to be reminded toward the clinicians proactively upon the practice protocol in CMU Hospital. Thus, all observed serum digoxin concentrations, especially those concentrations closed to the acceptable range i.e., 0.8 to 2.0 ng/ml, were tried out first to train the ANN models with the pre-specified potential variables. Then, only those observed concentrations, which were involved in the final ANN training models, were finally kept in the modeling dataset to be used further.

In the critical care settings, the dosage regimen for patients with severe illness is usually determined empirically. In addition to drawing blood samples from these neonatal patients, we collected the following information that was documented in the literature to be associated with PDA closure, heart failure progression and digoxin use for newborn infants [10–14] in order to explore its associations with the “observed serum digoxin concentrations”: demographic information (e.g., gender, postmenstrual age (PMA), total body weight (TBW)), disease status (e.g., being diagnosed with congestive heart failure (CHF), dilated cardiomyopathy (DCM), pulmonary hypertension (PH), v*entricular septal defect* (VSD)), and medications related to PDA closure or heart failure management (e.g., ibuprofen, captopril, furosemide).

### Confirmation of the appropriateness to perform traditional regression modeling

While there were various extents of correlations between drug clearance and PMA for different drugs during the first year of life [23], we examined the correlations between the volume of distribution (Vd) of digoxin that was normalized to bodyweight (i.e., /kg) and PMA for those enrolled newborn infants in the NICU who used digoxin and whose concentrations were expected to reach acceptable range first. Then, we examined the normality of these observed serum digoxin concentrations using a one-sample Kolmogorov-Smirnov test in order to explore the appropriateness of performing linear regressions on the modeling dataset. Further, we examined the bivariate correlations between observed digoxin concentrations and the pre-specified potential variables, i.e., patients with or without CHF, DCM, PH, VSD and medications used for PDA closure and/or heart failure management (including ibuprofen and captopril, furosemide) [10–14] on the modeling dataset to confirm the robustness of performing linear regression modeling.

### Model generation

The digoxin concentration models were generated using the following two methods on the modeling dataset: standard multivariable linear regressions (MLRs) and artificial neural networks (ANNs), whereas the initial 11 pre-specified potential variables were used as either independent variables for MLRs or input variables for ANNs, respectively.

#### Multivariable linear regression (MLR) model

We began to construct a 10-parameter digoxin linear regression model, regardless of extent of correlations between the observed digoxin concentrations and variables of interest, by using IBM® SPSS® statistics 25 with data from modeling samples. In particular, the dose, which was normalized to total body weight (i.e., /kg), was considered as a composite variable instead of two variables and was used, in addition to PMA and CHF, to avoid multicollinearity in MLR modeling. Then, we used the backward selection method to remove one variable at a time until the last model, which composed of common variables to predict digoxin concentrations (i.e., dose, total body weight, PMA, CHF) in population pharmacokinetics, was developed. Then, the prediction equation for each MLR model was prepared accordingly and subsequently used in the data obtained from a validation sample.

### ANN model

While ANN modeling is widely used to learn nonlinear mappings, and multicollinearity is not considered as a problem in training ANN models [24], both of the dose per kilogram (i.e., normalized to total body weight) and the patient’s weight were used to train the ANN models in this study. Then, an ANN model with a multilayer perceptron (MLP) was developed on the modeling dataset by using SPSS 25 [25]. The number of hidden layers, neuron number, and initial Lambda were modified constantly by repeated attempts in order to establish a model with better simulation results and avoid over-learning. Four-layered ANN architecture with 11 input variables was generated initially, where the following four layers were constructed: an input layer (input variables), two layers of hidden nodes and a single output layer. The ANN models are the mathematical equations that analyze the data in the input variables to compute an output variable and the bias neuron was incorporated in the input and hidden layers. We chose a standard backpropagation algorithm neural network, which is one of the most commonly used ANN architectures, for its robustness and excellent performance for pattern analysis of multivariable data. In the modeling, the network was trained 10 times, whereas new random sets of initial weights were used each time, and the model training was stopped whenever the maximum error between observed and predicted standardized values decreased to a value close to 1% was observed. After using reinitialized weights between neurons for each run several times, the results with the best fit between observations and the outputs predicted from training data were adopted as the optimized ANNs.

### Model accuracy and discriminant analysis for MLR and ANN modeling findings on the validation dataset

To test the derived ANN models and MLR models on an independent validation dataset, we used leave-one-out cross validation [26] to evaluate any over-fitting of the training data and tried to examine the differences between the prediction concentrations, which were compared with that of observed serum digoxin concentrations on the validation dataset. The four goodness-of-fit indexes, including the mean absolute deviation (MAD), mean absolute percent error (MAPE), mean square error (MSE), root mean squared error (RMSE) (i.e., the square root of the variance summation of the difference between observed and predicted outputs divided by the summation of the observed output variance [26]), were evaluated to measure the prediction accuracy. When the range of MAPE was less than 50%, the model was recognized as applicable to predict the serum digoxin concentrations. Smaller values of MAD, MAPE, MSE, and RMSE in the corresponding model were better and were considered as an optimal model. In addition, we performed receiver operating characteristic (ROC) curve analysis to classify the concentration as toxic or not (i.e., greater than or equal to 1.5 ng/ml upon the consensus between the corresponding practicing physicians and clinical pharmacists in this unit) when comparing the results obtained from MLR models or ANN models. The area under the ROC curve (AUC) referred to how well the prediction model could differentiate toxic and non-toxic levels, and we would expect to obtain better accuracy whenever the AUC range from 0.5 (random guess) to 1.0 (perfect accuracy) for the ROC curves [27].

Next, we performed discriminant analysis of MLR and ANN model findings for the predicted serum digoxin concentrations to investigate whether the patient’s predicted serum digoxin concentration would be equal to or above the toxic level (i.e., 1.5 ng/ml). A positive number indicated that the plasma concentration was equal to or above the toxic concentration, and a negative number indicated that the plasma concentration was below the toxic level. Once the results of model training became available, all the attempted models obtained from both MLR and ANN models were examined for their classifications based on the predicted concentrations as toxic or non-toxic, as compared to the observed serum digoxin concentrations, on the validation dataset: true positive (TP, correctly classified as ‘positive’), true negative (TN, correctly classified as ‘negative’), false positive (FP, incorrectly classified as ‘positive’), false negative (FN, incorrectly classified as ‘negative’), rate of correct prediction [RCP = (TP + TN)/(TP + TN + FP + FN)], sensitivity [SE = TP/(TP + FN), which infers the rate of correct predictions among all positive predictions], specificity [SP = TN/(TN + FP), which infers the rate of correct negative prediction among all negative predictions]. Specifically, the classifications of model performance were mainly evaluated its perdition rates by the following three criteria: SE, SP and RCP.

Overall, the final best model was determined based on the combined evaluation of accuracy (e.g., MSE, RMSE, MAD, MAPE), AUC for prediction discrimination, prediction rates (i.e., RCP, SE, SP), the importance and normalized importance, the correlations between observed and predicted digoxin concentrations by the best ANN model using the validation dataset, if this model did perform better than any of the MLR or ANN models.

## Results

After reviewing medical charts thoroughly and extracting needed data, we found originally 91 newborn infants contributed to 226 observations of serum digoxin concentrations in the first place. However, those who only contributed one observation of serum digoxin concentration, which was expected not reach steady state, or their concentrations were not within the acceptable therapeutic range (i.e., 0.8–2.0 ng/ml), especially those with extreme outliers of digoxin concentrations due to the neonatal patient’s critical conditions, were excluded from the training model. We eventually identified and used 139 observations of 71 newborn infants staying in NICU between April 1, 2013 to April 30, 2017 for model training and another 29 observations of 19 newborn infants staying in NICU between May 1, 2017 and December 31, 2017 for validation.

**Fig. 1:**
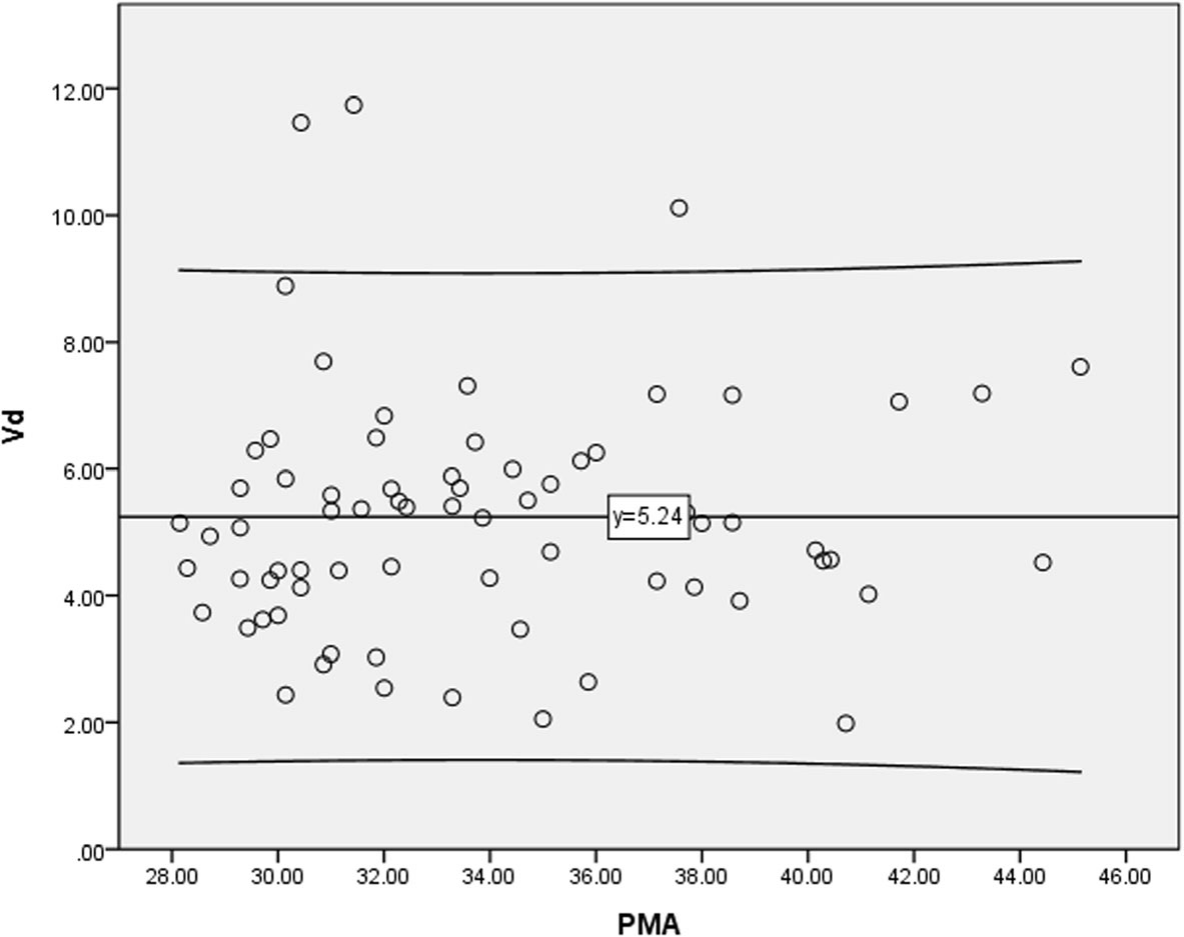
Digoxin volume distribution (Vd), which were normalized by bodyweight versus postmenstrual age (PMA) among all enrolled newborn infants in neonatal intensive care unit (one dose after steady-state; *n* = 71). Note: the line in the middle is the mean of Vd per total body weight (mean = 5.24 L/kg) and the upper and lower line are the upper and lower limits of 95% confidence interval for the mean Vd, respectively.

There were no statistically significant differences between the observed digoxin concentrations and pre-specified variables, except PH, in these two samples from modeling and validation datasets (Table 1). Of these recruited neonates, 58 (81.7%) and 13 (68.4%) were premature infants in the modeling and validation datasets, respectively, and their median PMA was 34 and 37, respectively. In all cases, TBW was approximately 1.73 kg. While the Vd of digoxin in full-term neonates is expected to be 7.5–10 L/kg [28], the mean Vd of digoxin for all enrolled neonates was relatively low and various across infants with different PMA (i.e., 5.24 L/kg in Fig. 1). Further, the distribution of observed digoxin concentrations for these critically ill infants was not normally distributed (*p* < 0.001 in Additional file 1: Table S1). There were no statistically significant correlations between the observed digoxin concentrations and the 10 pre-specified potential variables, except with or without PH (Additional file 1: Table S2). Thus, performing traditional linear regression modeling to predict the concentrations accordingly would violate some assumptions.

**Table 1:** Demographic, disease status and medication information among neonatal patients using digoxin on modeling dataset or validation dataset

	In modeling dataset	In validation dataset	*p*-value ^a^
Number of observations (male/female)	139 (81/58)	29 (13/16)	
Number of patients (male/female)	71 (40/31)	19 (7/12)	
Demographic			
Gender (Male/Female)	81/58	13/16	0.185
Postnatal age (week)	36.35 ± 8.08^b^, (34)^c^	38.41 ± 5.14^b^, (37)^c^	0.190
Total body weight (kg)	1.73 ± 0.90^b^, (1.44)^c^	1.88 ± 0.72^b^, (1.76)^c^	0.407
Diseases			
CHF	54 (38.8)	11 (37.9)	0.926
DCM	9 (6.5)	4 (13.8)	0.243
PH	11 (7.9)	7 (24.1)	0.010
VSD	16 (11.5)	9 (28.1)	0.072
Medications			
Ibuprofen	61 (38/23)	11 (3/8)	0.556
Captopril	6 (6/0)	4 (4/0)	0.072
Furosemide	24 (20/4)	5 (4/1)	0.997
Digoxin information			
Digoxin dose (mcg∙kg^−1^∙d^−1^)	5.90 ± 1.93, (5.19)	5.66 ± 1.81, (5.17)	0.542
Drug concentrations (ng/mL)	1.25 ± 0.38, (1.2)	1.14 ± 0.24, (1.06)	0.138

^a^. Nonparametric test by t-test for continuous data and Pearson’s Chi-Square test for categorized data; ^b^. Values are expressed as mean ± SD; ^c^. Median Value (IQR); *CHF* Congestive Heart Failure; *DCM* dilated cardiomyopathy; *PH* pulmonary hypertension; *VSD* Ventricular Septal Defect

Nevertheless, the 10-parameter MLR model and an 11-parameter ANN model was generated in the first place using the modeling dataset, respectively. The prediction performance applying findings obtained from the modeling dataset to the validation dataset were investigated with leave-one-variable-out cross validation as well. Tables 2, 3, and 4 show the ANN bootstrapping for a series of different input variables, which were analyzed using several specific classification techniques. The ranges of MAPE for all models were less than 50% and the highest two R^2^ were Model 3 or 4 (74.46, 73.82%, respectively) in Table 2. Although AUC ranges for all models were all more than 0.5, the highest two AUC values were Model 3 or 4 (0.738, 0.658, respectively), even if the confidence intervals of the corresponding AUC were overlapping across these nine proposed models (Table 3). Thus, the performance of the specified models were not significantly different from each other to differentiate the toxic concentration (i.e., ≥1.5 ng/ml) but Model 3 or 4 might the two better choices than the others.

**Table 2:** MSE, RMSE, MAD, MAPE of each ANN model between the observed serum digoxin concentrations and the corresponding predicting concentrations on validation dataset

Model	No. of parameters	Parameters	MAPE(%)	MSE	RMSE	MAD	R^2^(%)
1	11	All Variables	16.72	0.05	0.23	0.19	63.00
2	10	-Sex	15.88	0.05	0.22	0.17	65.17
3	9	-Sex-DCM	17.70	0.06	0.24	0.19	74.46
4	8	-Sex-DCM -PH	15.03	0.04	0.20	0.16	73.82
5	7	-Sex-DCM -PH -Captopril	21.41	0.09	0.30	0.24	57.30
6	6	-Sex-DCM -PH -Captopril -Furosemide	23.18	0.09	0.29	0.25	63.43
7	5	-Sex-DCM -PH -Captopril -Furosemide -VSD	25.16	0.11	0.33	0.26	46.37
8	4	-Sex-DCM -PH -Captopril -Furosemide -VSD -ibuprofen	24.68	0.10	0.31	0.26	44.43

“- “in the column of parameters refers to “exclude” that specific variable from the model 1, which contain all variables. *MAPE* Mean Absolute Percentage Error; *MSE* Mean Square Error; *RMSE* Root Mean Square Error; *MAD* Mean Absolute Deviation, *R*^*2*^*%* determination of coefficient

All 11 variables include: dose per total body weight, gender, postmenstrual age (PMA), Congestive heart failure (CHF), dilated cardiomyopathy (DCM), pulmonary hypertension (PH), V*entricular septal defect* (VSD), with captopril, with furosemide, with ibuprofen

*Common variables used in population pharmacokinetics were dose per total body weight, PMA, CHF

**Table 3:** Area under the curve (AUC) of the receiver operating characteristic (ROC) curves to differentiate toxicity concentration (i.e., equal and above 1.5 ng/ml) or not for each ANN model on validation dataset

Model	No. of parameters	Parameters	AUC	SE	Sig.	95% CI	
						Lower Bond	Upper Bond
1	11	All Variables	0.558	0.151	0.686	0.263	0.854
2	10	-Sex	0.658	0.152	0.273	0.361	0.956
3	9	-Sex-DCM	0.738	0.140	0.100	0.463	1.000
4	8	-Sex-DCM -PH	0.658	0.152	0.273	0.361	0.956
5	7	-Sex-DCM -PH -Captopril	0.575	0.147	0.603	0.286	0.864
6	6	-Sex-DCM -PH -Captopril -Furosemide	0.617	0.150	0.419	0.324	0.910
7	5	-Sex-DCM -PH -Captopril -Furosemide -VSD	0.633	0.141	0.356	0.357	0.909
8	4*	-Sex-DCM -PH -Captopril -Furosemide -VSD -ibuprofen	0.638	0.151	0.341	0.342	0.933

“- “in the column of parameters refers to “exclude” that specific variable from the model 1, which contain all variables. *AUC* area under the curve; *SE* standard error of AUC; *Sig.* significance of AUC finding

All 11 variables include: dose per total body weight, gender, postmenstrual age (PMA), Congestive heart failure (CHF), dilated cardiomyopathy (DCM), pulmonary hypertension (PH), V*entricular septal defect* (VSD), with captopril, with furosemide, with ibuprofen

*Common variables used in population pharmacokinetics were dose per total body weight, PMA, CHF

**Table 4:** Classification performance of prediction to differentiate toxicity concentrations (i.e., equal and above 1.5 ng/ml) or not, as compared to the observed serum digoxin concentrations, for each ANN model on validation dataset

Model	No. of parameters	Parameters	TP	TN	FP	FN	RCP(%)	SE(%)	SP(%)
1	11	All Variables	1	22	2	4	79.3	20	91.7
2	10	-Sex	2	22	2	3	82.8	40	91.7
3	9	-Sex-DCM	3	21	3	2	82.8	60	87.5
4	8	-Sex-DCM -PH	2	22	2	3	82.8	40	91.7
5	7	-Sex-DCM -PH -Captopril	2	18	6	3	69.0	40	75.0
6	6	-Sex-DCM -PH -Captopril -Furosemide	2	20	4	3	75.9	40	83.3
7	5	-Sex-DCM -PH -Captopril -Furosemide -VSD	3	16	8	2	65.5	60	66.7
8	4*	-Sex-DCM -PH -Captopril -Furosemide -VSD -ibuprofen	2	21	3	3	79.3	40	87.5

“- “in the column of parameters refers to “exclude” that specific variable from the model 1, which contain all variables. *TP* true positive (correctly classified to be ‘positive’); *TN* true negative (correctly classified to be ‘negative’); *FP* false positive (incorrectly classified to be ‘positive’); *FN* false negative (incorrectly classified to be ‘negative’), respectively; *RCP* rate of correct prediction; *SE* sensitivity; *SP* specificity

All variables include: dose per total body weight, weight, gender, postmenstrual age (PMA), Congestive heart failure (CHF), dilated cardiomyopathy (DCM), pulmonary hypertension (PH), V*entricular septal defect* (VSD), with captopril, with furosemide, with ibuprofen

*the common variables used in population pharmacokinetics were dose per total body weight, weight, PNA, CHF

When all the goodness-of-fit and prediction indexes (e.g., MSE, RMSE, MAD, MAPE, sensitivity, specificity, and AUC) were employed to evaluate the effectiveness of the prediction models in the validation dataset, finally, the Model 3 with 9 parameters stands out as having better performance (MAPE = 17.70%, *R*^2^ = 74.46%, AUC = 0.738, RCP = 82.8%, SE = 60%, SP = 87.5%) in comparison with the other ANN models and MLR models (i.e., eight-parameter model with MAPE = 16%, *R*^2^ = 54.9%, AUC = 0.9, RCP = 82.76%, SE = 16.67%, SP = 100% in Additional file 1: Tables S3, S4, and, S5). The Model 4 of 8-parameter ANN model and Model 4 of 7-parameter MLR model, respectively, were also better than the other models but relative less perfect than the Model 3 for both approaches. The TBW and PMA, other than “dose”, which was normalized to TBW, showed the greatest impact on the prediction of digoxin concentrations of all the pre-specified variables (Table 5).

**Table 5:** The importance of input variable for the best ANN model (Model 3 with 9 parameters) using validation dataset

	Importance	Normalized Importance
Dose/kg per dose	0.138	63.9%
TBW	0.216	100%
PMA	0.185	85.6%
PH	0.082	37.8%
CHF	0.066	30.7%
VSD	0.066	30.6%
Captopril	0.125	57.7%
Furosemide	0.050	23.3%
Ibuprofen	0.071	32.7%

Final model includes the following variables: dose/kg per dose, *TBW* total body weight; *PMA* postmenstrual age; *PH* pulmonary hypertension; *CHF* Congestive heart failure; *VSD* V*entricular septal defect*, with captopril, with furosemide, with ibuprofen

**Fig. 2:**
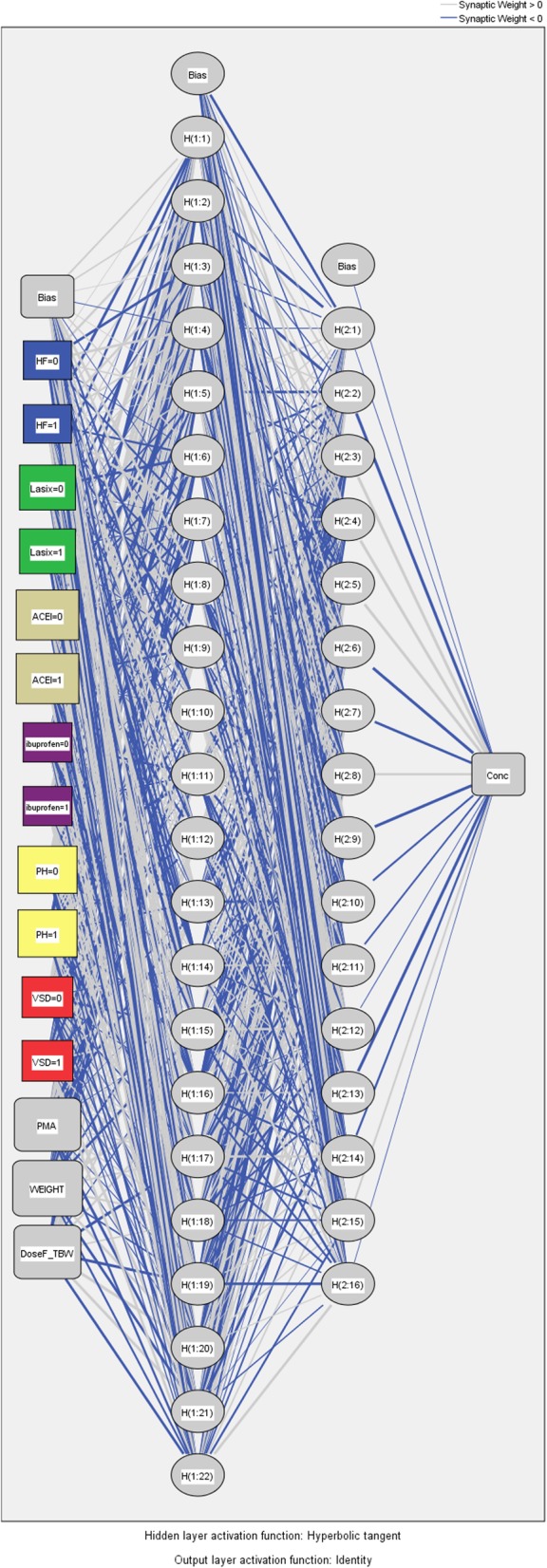
Multi-Layer Perceptron (MLP) model for the final best model (ANN Model 3 with 9-parameters) using modeling dataset

**Fig. 3:**
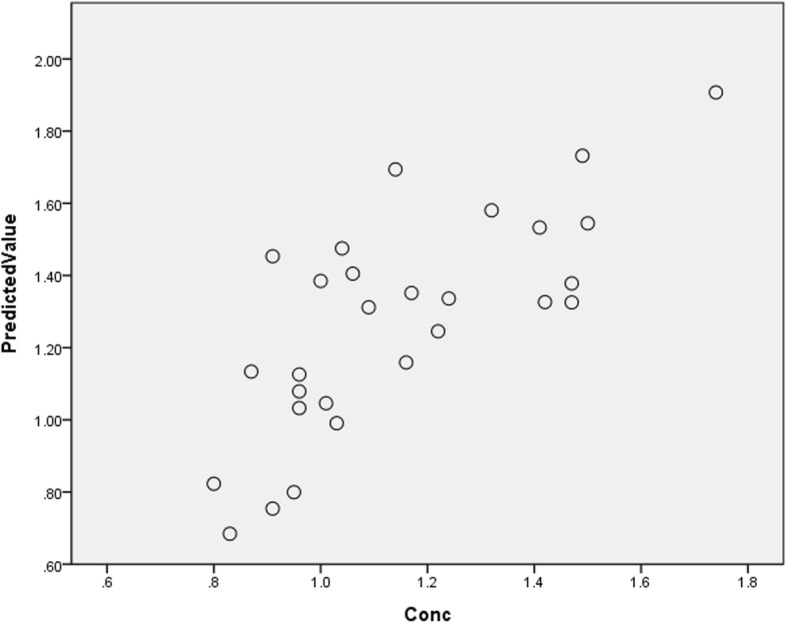
Correlation between observed and predicted digoxin concentrations by the best ANN model (ANN Model 3 with 9 parameters) using validation dataset. Correlation *r* = 0.743.

The final best nine-parameter ANN model consisting of the following three structural layers was identified as a better model (which was structured as that in Fig. 2) than the others: an input layer with 11 processing parameters (demographic, disease, and medications), two hidden layers with more parameters according to the number of input parameters (i.e., 22 and 16 parameters, respectively in layer two and layer three for the model with eight parameters), and an output layer with one processing element (predicted serum digoxin concentration). Consequently, the correlation between the observed and predicted serum digoxin concentrations on the validation dataset was 0.743 (Fig. 3), which met expectations.

## Discussion

Other than applying population PK approaches to predict digoxin concentrations and PK parameters for pediatric patients as has been done in Thailand and Japan [15–19], our study demonstrates that ANN modeling is a better alternative approach to predicting digoxin concentrations whenever drawing blood samples from critically ill newborn infants for therapeutic drug monitoring is very challenge. Especially, the nine-parameter ANN model is the final better model among all of the trained ANN and generated MLR models.

There is increasing interest in developing outcome prediction models using either traditional regression modeling and/or ANN modeling for pediatric patients, i.e., to predict survival in pediatric patients with trauma, as tools to assess medical quality, to evaluate reasonable resource allocation and research in medical care, and for use in comparing performance among institutions [21]. While the digoxin volume distribution in these critically ill newborn infants was relative low and also different from that of normal full-term neonates, it is understandable that the distribution of observed digoxin concentrations was not normally distributed and there were not strong enough correlations between the observed digoxin concentrations and the pre-specified potential variables. Such findings support that to perform traditional linear regression modeling and to predict the concentrations accordingly is not only problematic but also a big challenge.

While traditional statistical approaches assume linear correlations between the predicted digoxin concentrations and the pre-specified variables, MLR modeling is not a good approach because many statistical assumptions (non-linearity and multicollinearity) were violated. Nevertheless, we attempted to perform MLR modeling by ignoring these assumptions. Given that the ANN approach is one of the approaches recommended to overcome non-linearity concerns and multicollinearity of predictor variables [15], our study showed that ANN modeling did perform better than traditional regression modeling in predicting serum digoxin concentrations for critically ill newborn infants. This implies that ANN modeling is a better alternative choice of modeling approach, in agreement with other studies on the prediction of newborn infant growth [20], survival [21], and preterm birth [22].

While Hu et al. demonstrated the usefulness of data mining techniques (e.g., decision-tree-based and MLP) to predict the initial dose of digoxin using relevant variables for adults in Taiwan [19], our study showed that the ANN model with nine pre-specified variables (i.e., dose/kg, TBW, PMA, PH, CHF, VSD, captopril use, furosemide use, and ibuprofen use) exhibited better accuracy and prediction rate compared with the other ANN models. The eight-parameter model, excluding TBW, was also the better one among all MLR models. In other words, these pre-specified variables are the best choices as the inputs or independent variables to predict serum digoxin concentrations in ANN modeling or MLR modeling, respectively, for newborn infants with critical illness in the clinical practice setting in Taiwan. Chow et al. demonstrated that applying ANN modeling to incorporate demographic variables, weight, other diseases, and the dosage regimen to predict tobramycin concentrations for newborn infants resulted in findings similar to those obtained from a PK population using NONMEM® software [29]. All these evidences confirmed our findings that ANN is an alternative and useful modeling approach to predict digoxin concentrations or PK parameters without drawing blood samples from newborn infants. However, further research to compare and contrast our findings with analysis using population PK for digoxin concentrations in critically ill newborn infants is needed.

Our final nine-parameter ANN model to predict whether or not a toxic digoxin concentration is reached in newborn infants in their critical illness status has shown moderate sensitivity but better specificity, RCP and AUC for prediction discrimination (SE = 60%, SP = 87.5%, RCP = 82.8%, area under the ROC = 0.738). Such result was similar to the findings of a model developed by Hu et al. to predict the adult digoxin dose (RCP = 85.671% and area under the ROC = 0.813) [19]. Up to now, all these MLR or ANN models did not need to include the digoxin clearance of newborn infants, which is a necessary variable in traditraional PK approach or population PK software, e.g., NONMEM® software. Given that renal excretion functions are weak at birth but mature over a few months later [23], it is important to continually modify the drug dosage regimens designed for treating neonatal patients, including treatment with digoxin. That is because their developmental rates are rapid during the first few weeks and months of life. In contrast, our study revealed that TBW, PMA, PH, CHF, VSD, concomitant use of ibuprofen, captopril and/or furosemide, in addition to the dose variable, which was normalized to TBW, showed the greatest impact on digoxin concentrations when treating the newborn infants in critical illness. Of these variables, dose, TBW, and PMA, comparing to the other pre-specified variables, showed higher importance.

Some limitations of this study should be addressed when interpreting the findings. As in the other prediction study using MLR or ANN modeling, our datasets also have inherent imperfections in data collection and other factors. Further, the sample size was relatively small. Although Pasini demonstrated that ANN for small dataset analysis in complex medical areas is not a problem [30], we believe the generalizability of this study is still limited. That is because the modeling and validation dataset were derived from critically ill newborns in NICU in a single medical center, and these patients were relatively small, had lower volume distribution and had lower birth weight than in the other studies. A cross-validation study in other hospitals will be critical to confirm the validity of the better performance of ANN model in the future. Second, the ANN structure we chose to develop may not be sufficiently robust because the sensitivity of predicted toxic digoxin concentrations was relatively low even if the specificity and rate of correct prediction was high. One of the reasons could be that the power of the data search engine for model training might not be sufficient. Third, those outliers of observed serum digoxin concentrations in both directions were excluded for model training so that the findings limit its clinical use of ANN model for those patients who not reached the acceptable therapeutic range (i.e., 0.8–2.0 ng/ml) or those who were confronted with extreme higher concentrations due to some unknown conditions. Nevertheless, this is the first study to apply ANN modeling to predict serum digoxin concentrations in critically ill newborn infants. Fortunately, we obtained important input variables (including use of the other medications to close PDA or manage heart failure, e.g., ibuprofen, captopril, furosemide) to demonstrate that the nine-parameter ANN model was better calibrated than the other models, including MLR-derived models. Continuous application of this nine-parameter ANN model in the clinical practice settings for newborn infants with critical illness will be essential to validate its predictive value.

## Conclusion

While ANN models are better than MLR models, the nine-parameter ANN model is the best alternative to predict serum digoxin concentrations whenever blood samples from newborn infants for therapeutic drug monitoring are not available. This model has high specificity and better prediction accuracy to differentiate toxic from non-toxic predicted serum concentrations for newborn infants with critical illness than the other models. In this first study applying ANN and MLR modeling to predict serum digoxin concentrations especially in newborn infants with critical illness, we were fortunate to identify that the nine pre-specified input variables are important for training the ANN model as the better model relative to all other models. Further cross-validations using different samples of newborn infants in various disease states from different institutes are needed.

## Supplementary information

**Additional file 1: Table S1.** Table S1. Testing for normal distribution of concentrations for those whose concentrations were expected to reach acceptable range (i.e., 0.8–2.0 ng/dl and one person with one concentration) based on One Sample Kolmogorov-Smirnov Test. **Table S2**. Bivariate correlations between observed digoxn concentrations and the 10 pre-specified varables used for MLR modeling. **Table S3**. MSE, RMSE, MAD, MAPE of each MLR model between the observed serum digoxin concentrations and the corresponding predicting concentrations on validation dataset. **Table S4**. Area under the curve (AUC) of the receiver operating characteristic (ROC) curves to differentiate toxicity concentration (i.e., equal and above 1.5 ng/ml) or not for each MLR model on validation dataset. **Table S5**. Classification performance of prediction to differentiate toxicity concentrations (i.e., equal and above 1.5 ng/ml) or not, as compared to the observed serum digoxin concentrations, for each MLR model on validation dataset.

## Data Availability

The used data were retrospectively retrieved from electronic medical records of CMU Children’s Hospital and were transferred to analyzed data with de-identifiers under the requests and approval of IRB. Further, it was claimed that the data that support the findings of this study can only be accessed by the researchers and assistants in the team. Feel free to contact the corresponding authors regarding the availability of data and materials.

## Abbreviations

ANN: Artificial neural network
AUC: Area under the curve
CHF: Congestive heart failure
DCM: Dilated cardiomyopathy
FN: False negative
FP: False positive
MAD: Mean absolute deviation
MAPE: Mean absolute percent error
MLR: Multivariable linear regression
MSE: Mean square error
NICU: Neonatal Intensive Care Unit
PDA: Patent ductus arteriosus
PH: Pulmonary hypertension
PMA: Postmenstrual age
RCP: Rate of correct prediction
RMSE: Root mean squared error
ROC: Receiver operating characteristics
SE: Sensitivity
SP: Specificity
TBW: Total body weight
TN: True negative
TP: True positive
Vd: Volume of distribution
VSD: Ventricular septal defect
