# Supplementary material for: Predicting the serum digoxin concentrations of infants in the neonatal intensive care unit through an artificial neural network
Source: BMC Pediatr. 2019 Dec 27;19:517. doi: 10.1186/s12887-019-1895-7 (PMC6933639; doi:10.1186/s12887-019-1895-7)
Supplement: Supplementary file 1 — Additional file 1: Table S1. Table S1. Testing for normal distribution of concentrations for those whose concentrations were expected to reach acceptable range (i.e., 0.8–2.0 ng/dl and one person with one concentration) based on One Sample Kolmogorov-Smirnov Test. Table S2. Bivariate correlations between observed digoxn concentrations and the 10 pre-specified varables used for MLR modeling. Table S3. MSE, RMSE, MAD, MAPE of each MLR model between the observed serum digoxin concentrations and the corresponding predicting concentrations on validation dataset. Table S4. Area under the curve (AUC) of the receiver operating characteristic (ROC) curves to differentiate toxicity concentration (i.e., equal and above 1.5 ng/ml) or not for each MLR model on validation dataset. Table S5. Classification performance of prediction to differentiate toxicity concentrations (i.e., equal and above 1.5 ng/ml) or not, as compared to the observed serum digoxin concentrations, for each MLR model on validation dataset. [file 12887_2019_1895_MOESM1_ESM.docx]

**Supplements**

**Supplement Table 1. Testing for normal distribution of concentrations for those whose concentrations were expected to reach acceptable range (i.e., 0.8-2.0 ng/dl and one person with one concentration) based on One Sample Kolmogorov-Smirnov Test**

|  | | Concentration (ng/ml) |
| --- | --- | --- |
| N | | 71 |
| Normal Parameters | Mean | 1.192 |
|  | Std. Deviation | 0.3515 |
| Most Extreme Differences | Absolute | 0.186 |
|  | Positive | 0.186 |
|  | Negative | -0.110 |
| Kolmogorov-Smirnov Z | | 0.186 |
| Asymp. Sig. (2-tailed) | | **p<0.001** |

**Supplement Table 2. Bivariate correlations between observed digoxn concentrations and the 10 pre-specified varables used for MLR modeling**

|  | **Correlation coefficient** | **P value** |
| --- | --- | --- |
| Dose/kg per dose | 0.288 | 0.015 |
| TBW | -0.057 | 0.637 |
| PMA | -0.084 | 0.486 |
|  | **Concentration versus Concentration** | **P value** |
| CHF (Yes vs No) | 1.188±0.3591 vs 1.194±0.3513 | 0.942 |
| DCM (Yes vs No) | 1.360±0.3960 vs 1.179±0.3479 | 0.371 |
| PH (Yes vs No) | 1.562±0.4344 vs 1.158±0.3261 | 0.006* |
| VSD (Yes vs No) | 1.328±0.3751 vs 1.175±0.3477 | 0.303 |
| Ibuprofen (Yes vs No) | 1.230±0.3753 vs 1.160±0.3322 | 0.407 |
| Captopril (Yes vs No) | 1.240±0.6223 vs 1.190±0.3484 | 0.846 |
| Furosemide (Yes vs No) | 1.385±0.4304 vs 1.167±0.3363 | 0.099 |

All variables include: dose/kg per dose, gender, total body weight (TBW), postmenstrual age (PMA), Congestive heart failure (CHF), dilated cardiomyopathy (DCM), pulmonary hypertension (PH), Ventricular septal defect (VSD), with ibuprofen, with captopril, with furosemide

**Supplement Table 3. MSE, RMSE, MAD, MAPE of each MLR model between the observed serum digoxin concentrations and the corresponding predicting concentrations on validation dataset**

| **Model** | **No. of parameters** | **Parameters** | **MAPE(%)** | **MSE** | **RMSE** | **MAD** | **R^2^(%)** |
| --- | --- | --- | --- | --- | --- | --- | --- |
| 1 | 10 | All Variables | 32 | 0.18 | 0.43 | 0.37 | 64.0 |
| 2 | 9 | -Sex | 35 | 0.05 | 0.23 | 0.41 | 55.1 |
| **3** | **8** | **-Sex-DCM** | **16** | **0.05** | **0.23** | **0.28** | **54.9** |
| **4** | **7** | **-Sex-DCM -PH** | **17** | **0.05** | **0.23** | **0.18** | **44.4** |
| 5 | 6 | -Sex-DCM -PH -Captopril | 17 | 0.06 | 0.24 | 0.19 | 43.5 |
| 6 | 5 | -Sex-DCM -PH -Captopril -Furosemide | 17 | 0.05 | 0.23 | 0.20 | 21.4 |
| 7 | 4 | -Sex-DCM -PH -Captopril -Furosemide -VSD | 18 | 0.05 | 0.23 | 0.20 | 20.7 |
| 8 | 3 | -Sex-DCM -PH -Captopril -Furosemide -VSD -ibuprofen | 16 | 0.18 | 0.43 | 0.19 | 17.7 |

“-“ in the column of parameters refers to “exclude” that specific variable from the model 1, which contain all variables. MAPE=Mean Absolute Percentage Error; MSE=Mean Square Error; RMSE=Root Mean Square Error; MAD=Mean Absolute Deviation, R^2^ %= determination of coefficient

All variables include: dose per total body weight, gender, postmenstrual age (PMA), Congestive heart failure (CHF), dilated cardiomyopathy (DCM), pulmonary hypertension (PH), Ventricular septal defect (VSD), with captopril, with furosemide, with ibuprofen

*the common variables used in population pharmacokinetics were dose per total body weight, PMA, CHF

**Supplement Table 4. Area under the curve (AUC) of the receiver operating characteristic (ROC) curves to differentiate toxicity concentration (i.e., equal and above 1.5 ng/ml) or not for each MLR model on validation dataset**

| **Model** | **No. of parameters** | **Parameters** | **AUC** | **SE** | **Sig.** | **95% CI** | | |
| --- | --- | --- | --- | --- | --- | --- | --- | --- |
|  |  |  |  |  |  | **Lower Bond** | | **Upper Bond** |
| 1 | 10 | All Variables | 0.667 | 0.140 | 0.248 | 0.393 | 0.941 | |
| 2 | 9 | -Sex | 0.625 | 0.149 | 0.386 | 0.333 | 0.917 | |
| **3** | **8** | **-Sex-DCM** | **0.900** | **0.063** | **0.006** | **0.776** | **1.000** | |
| **4** | **7** | **-Sex-DCM -PH** | **0.717** | **0.165** | **0.133** | **0.393** | **1.000** | |
| 5 | 6 | -Sex-DCM -PH -Captopril | 0.717 | 0.165 | 0.133 | 0.393 | 1.000 | |
| 6 | 5 | -Sex-DCM -PH -Captopril -Furosemide | 0.650 | 0.123 | 0.299 | 0.408 | 0.892 | |
| 7 | 4 | -Sex-DCM -PH -Captopril -Furosemide -VSD | 0.658 | 0.136 | 0.273 | 0.391 | 0.925 | |
| 8 | 3* | -Sex-DCM -PH -Captopril -Furosemide -VSD -ibuprofen | 0.683 | 0.123 | 0.204 | 0.442 | 0.925 | |

“-“ in the column of parameters refers to “exclude” that specific variable from the model 1, which contain all variables. AUC= area under the curve; SE=standard error of AUC; Sig.= significance of AUC finding

All 11 variables include: dose per total body weight, gender, postmenstrual age (PMA), Congestive heart failure (CHF), dilated cardiomyopathy (DCM), pulmonary hypertension (PH), Ventricular septal defect (VSD), with captopril, with furosemide, with ibuprofen.

*Common variables used in population pharmacokinetics were dose per total body weight, PMA, CHF

**Supplement Table 5. Classification performance of prediction to differentiate toxicity concentrations (i.e., equal and above 1.5 ng/ml) or not, as compared to the observed serum digoxin concentrations, for each MLR model on validation dataset**

| **Model** | **No. of parameters** | **Parameters** | **TP** | **TN** | **FP** | **FN** | **RCP(%)** | **SE(%)** | **SP(%)** |
| --- | --- | --- | --- | --- | --- | --- | --- | --- | --- |
| 1 | 10 | All Variables | 0 | 24 | 0 | 5 | 82.76 | 0 | 100 |
| 2 | 9 | -Sex | 0 | 24 | 0 | 5 | 82.76 | 0 | 100 |
| **3** | **8** | **-Sex-DCM** | **1** | **23** | **0** | **5** | **82.76** | **16.67** | **100** |
| **4** | **7** | **-Sex-DCM -PH** | **1** | **24** | **0** | **4** | **86.21** | **20** | **100** |
| 5 | 6 | -Sex-DCM -PH -Captopril | 1 | 24 | 0 | 4 | 86.21 | 20 | 100 |
| 6 | 5 | -Sex-DCM -PH -Captopril -Furosemide | 1 | 25 | 0 | 3 | 89.66 | 25 | 100 |
| 7 | 4 | -Sex-DCM -PH -Captopril -Furosemide -VSD | 0 | 24 | 0 | 5 | 82.76 | 0 | 100 |
| 8 | 3* | -Sex-DCM -PH -Captopril -Furosemide -VSD -ibuprofen | 0 | 24 | 0 | 5 | 82.76 | 0 | 100 |

“-“ in the column of parameters refers to “exclude” that specific variable from the model 1, which contain all variables. TP, true positive (correctly classified to be ‘positive’); TN, true negative (correctly classified to be ‘negative’); FP, false positive (incorrectly classified to be ‘positive’); FN, false negative (incorrectly classified to be ‘negative’), respectively; RCP, rate of correct prediction; SE, sensitivity; SP, specificity.

All variables include: dose/kg per dose, gender, postmenstrual age (PMA), Congestive heart failure (CHF), dilated cardiomyopathy (DCM), pulmonary hypertension (PH), Ventricular septal defect (VSD), with captopril, with furosemide, with ibuprofen

*the common variables used in population pharmacokinetics were Dose, TBW, PMA, CHF
